# Personality Functioning in Current Epilepsy Patients and People Recovered from Epilepsy

**DOI:** 10.3390/reports6020021

**Published:** 2023-05-01

**Authors:** Weixi Kang, Antonio Malvaso

**Affiliations:** 1 UK DRI Care Research and Technology Centre, Department of Brain Sciences, Imperial College London, London W12 0BZ, UK; 2 IRCCS “C. Mondino” Foundation, National Neurological Institute, Department of Brain and Behavioral Sciences, University of Pavia, 27100 Pavia, Italy; malvaso.antonio@libero.it

**Keywords:** personality, epilepsy, neuroticism, openness, conscientiousness, extraversion

## Abstract

The aim of the current study is to examine the effect of epilepsy recovery on personality traits by analyzing data from a large representative cohort from the United Kingdom. This study used data from Understanding Society: the UK Household Longitudinal Study (UKHLS). A predictive normative modelling approach along with one-sample t-tests were used to analyze the personality trait differences between 190 current epilepsy patients (40% male) with a mean age of 43.95 ± 15.68 years and 102 previous epilepsy patients (45.1% male) with a mean age of 47.50 ± 15.27 years. People who recovered from epilepsy had lower Neuroticism but higher Openness, Conscientiousness, and Extraversion scores than people who did not recover from epilepsy after controlling for demographic covariates, which can be potentially explained by the psychophysiological pathways. Health professionals can make better post-discharge plans based on personality traits as a result of epilepsy recovery.

## 1. Introduction

Epilepsy is one of the most common and serious neurological diseases. It affects around 50 million people around the world [1]. The term “personality” refers to the basic level of individual differences in terms of the way people feel, think, and behave. Although personality traits are generally considered stable across a lifetime, recent studies have shown that life events, stressors, and challenges can lead to changes in personality traits [2]. For instance, the presence of a chronological condition may require patients to think about their identity, values, and plans for the future. For these reasons, some scholars have conceptualized chronic illness as a biographical disruption [3]. Moreover, personality traits are also closely related to brain structure and functions, which may provide another reason why epilepsy can affect personality traits as measured by the Big Five, i.e., Neuroticism, Agreeableness, Openness, Conscientiousness, and Extraversion.

Studies have investigated the impact of epilepsy on personality traits, specifically, the Big Five traits, and have yielded significant findings. Epilepsy patients were found to have higher levels of Neuroticism compared to healthy controls [4,5,6,7,8,9,10]. For instance, Wilson et al. (2009) conducted a two-year prospective longitudinal study that included sixty people with chronic epilepsy. They found that individuals with epilepsy had higher Neuroticism scores compared to controls, particularly those who experienced epilepsy during the self-definable phase of adolescence [9]. Similarly, Findikli et al. (2016) compared 78 epilepsy patients (33 ± 12.82 years; 58.9% female) to 76 healthy volunteers (30.76 ± 6.44 years; 60.5% female) matched for age and gender and observed significantly higher Neuroticism scores in the epilepsy group [6]. High levels of Neuroticism have been shown to negatively impact the quality of life for epilepsy patients [6].

In addition, epilepsy patients were found to have lower Openness scores than healthy controls [5,8] and individuals with non-epileptic seizures [10]. Leong et al. (2019) conducted research that included 122 patients with epileptic seizures (38.87 ± 14.73 years, 65.57% women), 90 patients with psychogenic non-epileptic seizures (35.28 ± 12.70 years, 68.89% women), 14 patients with both pathologies (36.21 ± 11.91 years, 78.57% women), and 79 patients for whom a diagnosis could not be determined (43.13 ± 18.37 years, 62.03% women). Their study found that epilepsy is associated with lower Openness scores than psychogenic seizures, and epilepsy patients have less Openness scores than the general population [10]. Similarly, Bonet et al. (2019) compared 31 healthy controls (32.8 ± 8.9 years; 41% women) with 67 people with temporal lobe epilepsy (TLE) (34.6 ± 9.5 years; 67% women) and found that TLE patients exhibited lower Openness scores [5].

Although previous studies looked at how epilepsy could affect personality traits, those studies did not look at the differences in personality traits between people currently with epilepsy and people who have recovered from epilepsy, which is of great importance, given that studies have shown that personality traits are related to various psychosocial and health-related outcomes such as health, relationships, work, and well-being. Understanding the effect of epilepsy recovery can help clinicians to make better post-discharge plans. Thus, the aim of the current study is to examine the effect of epilepsy recovery on personality traits by analyzing data from a large representative cohort from the United Kingdom.

## 2. Methods

### 2.1. Data

This study used data from Understanding Society: the UK Household Longitudinal Study (UKHLS), which has been collecting annual information from the original sample of UK households since 1991 (when it was previously known as The British Household Panel Study (BHPS) [11]. This dataset is publicly available at https://www.understandingsociety.ac.uk (accessed on 1 September 2022). All data collections have been approved by the University of Essex Ethical Committee. Participants completed informed consent before participating in these studies. Participants completed the demographics and epilepsy question at Wave 1, which was collected between 2009 and 2010; they also completed personality measures at Wave 3, which was collected between 2011 and 2012. Participants with any missing variables of interest were removed from further analyses. Thus, there were 190 participants with a mean age of 43.95 ± 15.68 years who indicated that they were current epilepsy patients and 102 with a mean age of 47.50 ± 15.27 years who indicated that they have been clinically diagnosed with epilepsy but no longer have it now.

### 2.2. Measures

#### 2.2.1. Personality Traits

Personality was measured using the 15-item version of the Big Five Inventory, with a Likert scale ranging from 1 (“disagree strongly”) to 5 (“agree strongly”). Scores were reverse coded when appropriate. The exact set of questions used can be found at https://www.understandingsociety.ac.uk/documentation/mainstage/dataset-documentation/term/personality-traits?search_api_views_fulltext (accessed on 1 September 2022). Mean scores were used for each of these traits. All personality scores were standardized (mean = 0, SD = 1) before further analysis.

#### 2.2.2. Epilepsy

Self-reported epilepsy is a valid measure to identify epilepsy at a population level (e.g., [12]). Among people who have indicated that they have been clinically diagnosed with epilepsy, they additionally answered the question “Do you still have epilepsy?” to indicate if they still have epilepsy.

#### 2.2.3. Demographic Controls

Demographic controls in the model include age, sex, monthly income, highest educational qualification, legal marital status, and residence.

### 2.3. Analysis

A predictive normative modelling approach was used to analyze the data. First, five generalized linear models were trained by taking demographics from current epilepsy patients as the predictors and their personality trait scores as the predicted variables, respectively. Then, demographics from people who have recovered from epilepsy were taken into the model as the predictors to predict the expected scores in people who have recovered from epilepsy. Finally, one-sample t-tests were conducted to determine the differences between the predicted and actual personality scores in people who had recovered from epilepsy. This predictive normative modeling approach could control for any unbalance demographic characteristics that may affect the results.

## 3. Results

Descriptive statistics for people with epilepsy and recovered from epilepsy can be found in Table 1. There was a main effect of sex (F(1, 183) = 8.61, *p* < 0.01) on Neuroticism, a main effect of age (F(1, 183) = 6.78, *p* < 0.05) and sex (F(1, 183) = 4.77, *p* < 0.05) on Agreeableness, and a main effect of monthly income (F(1, 183) = 11.38, *p* < 0.001) on people who were current epilepsy patients. Demographic variables that were not mentioned were not significant.

## 4. Discussion

The aim of the current study was to compare the differences in personality traits in people who had recovered from epilepsy and people with epilepsy currently. By using a predictive normative modeling approach along with one-sample t-tests on data from UKHLS, the current study found that those who had recovered from epilepsy had lower Neuroticism but higher Openness, Conscientiousness, and Extraversion scores than people who had not recovered from epilepsy after controlling for demographic covariates.

The finding that patients who had recovered from epilepsy showed significantly lower Neuroticism but higher Openness than those who had not recovered aligns with the broader literature: epilepsy has been associated with higher Neuroticism [4,5,6,7,8,9,10] and lower Openness compared with healthy controls [5,8], suggesting that recovery may be linked to a personality profile closer to that observed in non-clinical samples.

The findings from the current study may be explained by the psychophysiological pathways that underlie the observed results. For instance, a previous study showed that an increase in Neuroticism is significantly associated with hypersynchrony between the right hippocampus and Brodmann area 9 (i.e., a region of the prefrontal cortex), as well as with the connection between the right hippocampus and Brodmann area 47 (i.e., the anterior frontal operculum) [5]. Additionally, higher levels of Neuroticism are associated with decreased grey matter volume, which is seen only in the left hemisphere for cortical regions that include the precentral gyrus, rostral superior and middle frontal gyri, superior temporal gyrus, anterior insula, lateral parietal-occipital cortex, fusiform gyrus, and precuneus [5]. These results suggest that a unitary etiology exclusive to the mesial temporal lobe is too narrow to be considered, and that locations beyond the temporal lobe, including but not limited to those mentioned, could contribute to changes in a subset of basic personality traits.

Various studies have indicated that Neuroticism is linked to insula, amygdala, and anterior cingulate activities at rest or in reaction to unpleasant or new stimuli [13,14,15,16,17,18,19]. Furthermore, Neuroticism has been associated with decreased volume and neural activities in the medial prefrontal cortex, both of which are indicative of poor emotion regulation [20,21,22]. These findings are consistent with observations made by Bonet et al. (2019) concerning patients with temporal lobe epilepsy (TLE), who had greater levels of Neuroticism than healthy controls. Since insula, amygdala, anterior cingulate, and medial prefrontal cortex are impaired in TLE, it is possible that after recovery, these regions would also recover, resulting in lower Neuroticism scores for individuals who have recovered from epilepsy.

Openness is thought to represent interaction with abstract or intellectual knowledge and engagement with sensory information [23] and is considered the only Big Five component that consistently correlates positively with cognitive and working memory capacity [23,24]. However, research using functional magnetic resonance imaging (fMRI) has found no relationship between Openness and brain activity during a challenging working memory task in the left frontal cortex and posterior medial frontal cortex, two areas of the prefrontal cortex [24]. Nonetheless, Openness is likely related to prefrontal cortex activities, which are crucial for planning and adhering to complicated regulations in humans [25,26]. As previously mentioned, Leong et al. (2019) found that epilepsy is related to lower Openness scores than those of the general population [10]. Additionally, although not significant, Bonet et al. (2019) demonstrated that TLE patients showed lower Openness values. In individuals who have recovered from epilepsy, abstract or intellectual knowledge and engagement with sensory information, partially related to the prefrontal cortex, might be improved, resulting in higher Openness scores according to our findings.

Conscientiousness is believed to be associated with the functioning of the prefrontal cortex [25,26]. Specifically, a structural MRI study found that individuals with higher Conscientiousness scores had a larger volume of the middle frontal gyrus in the lateral prefrontal cortex, which is responsible for maintaining goal-relevant information in working memory and carrying out planned actions based on abstract rules [25]. Since epilepsy can cause impairment to the prefrontal cortex [27,28], it stands to reason that Conscientiousness may be impacted as well. We observed that individuals who had recovered from epilepsy scored higher on Conscientiousness than those who still had the condition, which is consistent with the idea that prefrontal cortex function may improve after epilepsy recovery.

Moreover, studies using functional MRI have suggested that Extraversion is positively correlated with brain activity in several regions, including the amygdala, medial orbitofrontal cortex, nucleus accumbens, and striatum [29,30,31,32]. Multiple structural MRI studies have also linked Extraversion to a larger volume of the medial orbitofrontal cortex, a region involved in coding the value of rewards [22,33,34]. Our findings indicate that individuals who had recovered from epilepsy had higher Extraversion scores than those who still had the condition; this could be explained by the recovery of brain regions such as the amygdala, medial orbitofrontal cortex, nucleus accumbens, striatum, and medial orbitofrontal cortex.

The results from the current study may encourage future neuroscientific endeavors trying to clarify the underlying neural pathways between epilepsy recovery and personality traits. Despite the strength of the current study, there are also some limitations. First, the current study is a cross-sectional and between-subject comparison, which cannot establish a causal effect. Second, all the data were self-reported, which cannot avoid self-reporting bias. Third, a person who is seizure free on medication could potentially answer that they no longer have epilepsy, and the severity of seizures (several vs chronic) was not controlled, which may make it hard to rule out the effect of seizures on personality [35].

Finally, the information about sub-type of epilepsy was not available, which also could be a modifying factor. Future research should focus on comparing the differences in personality traits of pre- and post-epilepsy recovery on the same subject and measuring epilepsy more objectively and comprehensively to check whether the findings from the current study are still supported.

## 5. Conclusions

In conclusion, the current study found that those who had recovered from epilepsy had lower Neuroticism but higher Openness, Conscientiousness, and Extraversion scores than people who had not recovered from epilepsy after controlling for demographic covariates. These findings have some clinical implications. Specifically, health professionals can make better post-discharge plans based on personality traits as a result of epilepsy recovery.

## Figures and Tables

**Figure 1:**
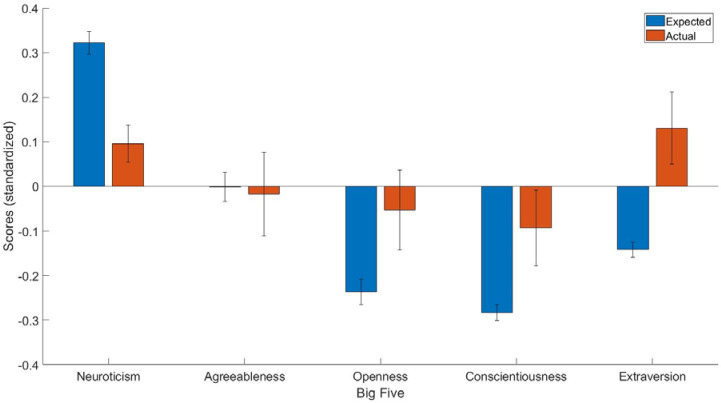
The expected and predicted Big Five scores for people who had recovered from epilepsy. The blue bar represents expected scores in people who had recovered from epilepsy, whereas the red bar represents the actual personality scores in people who had recovered from epilepsy. Y axis = standardized scores.

**Table 1:** Descriptive statistics for people currently with epilepsy and recovered from epilepsy.

	Current Epilepsy Patients		People who Recovered from Epilepsy	
	Mean	S.D.	Mean	S.D.
Age	43.95	15.68	45.70	15.27
Monthly income	1238.25	1180.45	1144.04	705.28
Neuroticism	4.10	1.63	3.69	1.46
Agreeableness	5.61	1.22	5.66	1.04
Openness	4.24	1.51	4.48	1.19
Conscientiousness	5.15	1.23	5.42	1.01
Extraversion	4.43	1.33	4.76	1.22
	N	%	N	%
**Highest educational qualification**				
Below college	76	40.00	46	45.10
College	114	60.00	56	54.90
**Legal marital status**				
Single	148	77.89	82	80.39
Married	42	22.11	20	19.61
**Residence**				
Urban	105	55.26	43	42.16
Rural	85	44.74	59	57.84

The main finding was that people who recovered from epilepsy had lower Neuroticism (t(101) = −2.47, *p* < 0.05, Cohen’s d = −0.23, 95% C.I. [−0.41, −0.04]) but higher Openness (t(101) = 2.02, *p* < 0.05, Cohen’s d = 0.18, 95% C.I. [0.003, 0.36]), Conscientiousness (t(101) = 2.36, *p* < 0.05, Cohen’s d = 0.19, [0.03, 0.35]), and Extraversion (t(101) = 3.16, *p* < 0.01, Cohen’s d = 0.27, 95% C.I. [0.10, 0.44]) scores than people who did not recover from epilepsy (Figure 1).

## Data Availability

This data can be found here: https://www.understandingsociety.ac.uk (accessed on 1 September 2022).

## References

[1] World Health Organization (WHO) Epilepsy. https://www.who.int/news-room/fact-sheets/detail/epilepsy#:~:text=Epilepsy%20is%20a%20chronic%20noncommunicable,%2D%20and%20middle%2Dincome%20countries.

[2] Roberts B.W., Walton K.E., Viechtbauer W. (2006). Patterns of mean-level change in personality traits across the life course: A meta-analysis of longitudinal studies. Psychol. Bull..

[3] Williams S.J. (2000). Chronic illness as biographical disruption or biographical disruption as chronic illness? Reflections on a core concept. Sociol. Health Illn..

[4] Rassart J., Luyckx K., Verdyck L., Mijnster T., Mark R.E. (2020). Personality functioning in adults with refractory epilepsy and community adults: Implications for health-related quality of life. Epilepsy Res..

[5] Bonet C.N.R., Hermann B., Cook C.J., Hwang G., Dabbs K., Nair V., Forseth C., Mathis J., Allen L., Almane D.N. (2019). Neuroanatomical correlates of personality traits in temporal lobe epilepsy: Findings from the Epilepsy Connectome Project. Epilepsy Behav..

[6] Findikli E., Izci F., Camkurt M.A., Tuncel D., Sahin M.C., Kuran M.Y., Demirhan S.Ö. (2016). Eysenck personality characteristics of epilepsy patients and its effect on quality of life. J. Mood Dis..

[7] Shehata G.A., Bateh A.E.A.M. (2009). Cognitive function, mood, behavioral aspects, and personality traits of adult males with idiopathic epilepsy. Epilepsy Behav..

[8] Wang X., Lv Y., Zhang W., Meng H. (2018). Cognitive impairment and personality traits in epilepsy: Characterization and risk factor analysis. J. Nerv. Ment. Dis..

[9] Wilson S.J., Wrench J.M., McIntosh A.M., Bladin P.F., Berkovic S.F. (2009). Personality development in the context of intractable epilepsy. Arch. Neurol..

[10] Leong M., Wang A.D., Trainor D., Johnstone B., Rayner G., Kalincik T., Roos I., Kwan P., O’brien T.J., Velakoulis D. (2019). Personality profiles differ between patients with epileptic seizures and patients with psychogenic non-epileptic seizures. Seizure.

[11] University of Essex, Institute for Social and Economic Research (2022). Understanding Society: Waves 1–11, 2009–2020 and Harmonised BHPS: Waves 1–18, 1991–2009.

[12] Brooks D.R., Avetisyan R., Jarrett K.M., Hanchate A., Shapiro G.D., Pugh M.J., Berlowitz D., Thurman D., Montouris G., Kazis L.E. (2012). Validation of self-reported epilepsy for purposes of community surveillance. Epilepsy Behav..

[13] Deckersbach T., Miller K.K., Klibanski A., Fischman A., Dougherty D.D., Blais M.A., Herzog D.B., Rauch S.L. (2006). Regional cerebral brain metabolism correlates of Neuroticism and Extraversion. Depress. Anxiety.

[14] Eisenberger N.I., Lieberman M.D., Satpute A.B. (2005). Personality from a controlled processing perspective: An fMRI study of neuroticism, extraversion, and self-consciousness. Cogn. Affect. Behav. Neurosci..

[15] Etkin A., Klemenhagen K.C., Dudman J.T., Rogan M.T., Hen R., Kandel E.R., Hirsch J. (2004). Individual differences in trait anxiety predict the response of the basolateral amygdala to unconsciously processed fearful faces. Neuron.

[16] Cools R., Calder A.J., Lawrence A.D., Clark L., Bullmore E., Robbins T.W. (2005). Individual differences in threat sensitivity predict serotonergic modulation of amygdala response to fearful faces. Psychopharmacology.

[17] Haas B.W., Omura K., Constable T., Canli T. (2007). Emotional conflict and Neuroticism: Personalitydependent activation in the amygdala and subgenual anterior cingulate. Behav. Neurosci..

[18] Keightley M.L., Seminowicz D.A., Bagby R.M., Costa P.T., Fossati P., Mayberg H.S. (2003). Personality influences limbic-cortical interactions during sad mood. NeuroImage.

[19] Reuter M., Stark R., Henning J., Walter B., Kirsch P., Schienle A., Vaitl D. (2004). Personality and emotion: Test of Gray’s personality theory by means of an fMRI study. Behav. Neurosci..

[20] Williams L.M., Brown K.J., Palmer D., Liddell B.J., Kemp A.H., Olivieri G., Peduto A., Gordonl E. (2006). The mellow years?: Neural basis of improving emotional stability over age. J. Neurosci..

[21] Haas B.W., Constable R.T., Canli T. (2008). Stop the sadness: Neuroticism is associated with sustained medial prefrontal cortex response to emotional facial expressions. Neuroimage.

[22] DeYoung C.G., Hirsh J.B., Shane M.S., Papademetris X., Rajeevan N., Gray J.R. (2010). Testing predictions from personality neuroscience: Brain structure and the big five. Psychol. Sci..

[23] DeYoung C.G., Peterson J.B., Higgins D.M. (2005). Sources of Openness/Intellect: Cognitive and neuropsychological correlates of the fifth factor of personality. J. Pers..

[24] DeYoung C.G., Gray J.R., Corr P.J., Matthews G. (2009). Personality neuroscience: Explaining individual differences in affect, behavior, and cognition. The Cambridge Handbook of Personality Psychology.

[25] Bunge S.A., Zelazo P.D. (2006). A brain-based account of the development of rule use in childhood. Curr. Dir. Psychol. Sci..

[26] Miller E.K., Cohen J.D. (2001). An integrative theory of prefrontal cortex function. Annu. Rev. Neurosci..

[27] Jokeit H., Seitz R.J., Markowitsch H.J., Neumann N., Witte O.W., Ebner A. (1997). Prefrontal asymmetric interictal glucose hypometabolism and cognitive impairment in patients with temporal lobe epilepsy. Brain J. Neurol..

[28] Stretton J., Thompson P.J. (2012). Frontal lobe function in temporal lobe epilepsy. Epilepsy Res..

[29] Canli T., Sivers I., Whitfield S.L., Gotlib I.H., Gabrieli J.D.E. (2002). Amygdala response to happy faces as a function of Extraversion. Science.

[30] Canli T., Zhao Z., Desmond J.E., Kang E., Gross J., Gabrieli J.D.E. (2001). An fMRI study of personality influences on brain reactivity to emotional stimuli. Behav. Neurosci..

[31] Cohen M.X., Young J., Baek J.-M., Kessler C., Ranganath C. (2005). Individual differences in extraversion and dopamine genetics predict neural reward responses. Cogn. Brain Res..

[32] Mobbs D., Hagan C.C., Azim E., Menon V., Reiss A.L. (2005). Personality predicts activity in reward and emotional regions associated with humor. Proc. Natl. Acad. Sci. USA.

[33] Omura K., Constable R.T., Canli T. (2005). Amygdala gray matter concentration is associated with extraversion and neuroticism. NeuroReport.

[34] Rauch S.L., Milad M.R., Orr S.P., Quinn B.T., Fischl B., Pitman R.K. (2005). Orbitofrontal thickness, retention of fear extinction, and extraversion. NeuroReport.

[35] Iurina E., Bailles E., Pintor L. (2021). Personality changes in patients with refractory epilepsy after surgical treatment: A systematic review. Seizure.

