# Correction: Kang, W.; Malvaso, A. Personality Functioning in Current Epilepsy Patients and People Recovered from Epilepsy. *Reports* 2023, *6*, 21

**DOI:** 10.3390/reports9010087

**Published:** 2026-03-16

**Authors:** Weixi Kang, Antonio Malvaso

**Affiliations:** 1 School of Arts and Humanities, Tung Wah College, 136A, Nathan Road, Kowloon, Hong Kong; 2 IRCCS “C. Mondino” Foundation, National Neurological Institute, Department of Brain and Behavioral Sciences, University of Pavia, 27100 Pavia, Italy

**Text Correction**

There was an error in the original publication [1]. In Section 4 (Discussion), Paragraph 2, the text incorrectly stated that patients who had recovered from epilepsy had higher Neuroticism (and Openness) than those who had not recovered; however, the results indicate lower Neuroticism but higher Openness in the recovered group.

A correction has been made to Section 4. Discussion, Paragraph 2:

The finding that patients who had recovered from epilepsy showed significantly lower Neuroticism but higher Openness than those who had not recovered aligns with the broader literature: epilepsy has been associated with higher Neuroticism [4–10] and lower Openness compared with healthy controls [5,8], suggesting that recovery may be linked to a personality profile closer to that observed in non-clinical samples.

The authors state that the scientific conclusions are unaffected. This correction was approved by the Academic Editor. The original publication has also been updated.

## References

[1] Kang W., Malvaso A. (2023). Personality Functioning in Current Epilepsy Patients and People Recovered from Epilepsy. Reports.

